# Systematic analysis of *CNGCs* in cotton and the positive role of *GhCNGC32* and *GhCNGC35* in salt tolerance

**DOI:** 10.1186/s12864-022-08800-5

**Published:** 2022-08-05

**Authors:** Zhengying Lu, Guo Yin, Mao Chai, Lu Sun, Hengling Wei, Jie Chen, Yufeng Yang, Xiaokang Fu, Shiyun Li

**Affiliations:** 1 Handan Academy of Agricultural Sciences, Handan, China; 2 State Key Laboratory of Cotton Biology, Institute of Cotton Research, Chinese Academy of Agricultural Sciences (CAAS), Anyang, China

**Keywords:** Cotton, *GhCNGC* genes, Salt tolerance, Abscisic acid, Abiotic stress

## Abstract

**Background:**

Cyclic nucleotide-gated ion channels (*CNGCs*) are calcium-permeable channels that participate in a variety of biological functions, such as signaling pathways, plant development, and environmental stress and stimulus responses. Nevertheless, there have been few studies on *CNGC* gene family in cotton.

**Results:**

In this study, a total of 114 *CNGC* genes were identified from the genomes of 4 cotton species. These genes clustered into 5 main groups: I, II, III, IVa, and IVb. Gene structure and protein motif analysis showed that *CNGCs* on the same branch were highly conserved. In addition, collinearity analysis showed that the *CNGC* gene family had expanded mainly by whole-genome duplication (WGD). Promoter analysis of the *GhCNGCs* showed that there were a large number of cis-acting elements related to abscisic acid (ABA). Combination of transcriptome data and the results of quantitative RT–PCR (qRT–PCR) analysis revealed that some *GhCNGC* genes were induced in response to salt and drought stress and to exogenous ABA. Virus-induced gene silencing (VIGS) experiments showed that the silencing of the *GhCNGC32* and *GhCNGC35* genes decreased the salt tolerance of cotton plants (TRV:00). Specifically, physiological indexes showed that the malondialdehyde (MDA) content in gene-silenced plants (TRV:*GhCNGC32* and TRV:*GhCNGC35*) increased significantly under salt stress but that the peroxidase (POD) activity decreased. After salt stress, the expression level of ABA-related genes increased significantly, indicating that salt stress can trigger the ABA signal regulatory mechanism.

**Conclusions:**

we comprehensively analyzed *CNGC* genes in four cotton species, and found that *GhCNGC32* and *GhCNGC35* genes play an important role in cotton salt tolerance. These results laid a foundation for the subsequent study of the involvement of cotton *CNGC* genes in salt tolerance.

**Supplementary Information:**

The online version contains supplementary material available at 10.1186/s12864-022-08800-5.

## Background

The coordinated control of Ca^2+^ signaling is essential for the development of eukaryotes [1]. Members of the cyclic nucleotide-gated channel (*CNGC*) family mediate Ca^2+^ influx in plant cells [2, 3]. In plants, CNGCs are composed of six transmembrane (TM) domains and one pore region between the fifth and sixth TM domains. The cyclic nucleotide-binding domain (CNBD) is a highly conserved region and has a phosphate-binding cassette (PBC) and a hinge region [4]. The CNBD is highly conserved and carries a plant CNGC-specific motif spanning the PBC and hinge region. This motif is unique to CNGCs and is hence recognized as an authentic means to identify plant CNGCs [5–7].

Abiotic stress is an important environmental condition that decreases plant growth, productivity and quality [8]. Soil salinity is one of the most frequent types of abiotic stress [9]. Thirty crop species provide 90% of food worldwide, and most of these species experience severe yield losses under moderate salinity [10]. Studies have shown that plant *CNGCs* participate in various processes related to growth and development [11, 12]. In particular, the plant *CNGC* gene family plays an important role in the response to abiotic stress and related signal transduction [13–15]. The *CNGC* family members compose a group of nonselective cation channels and enable the uptake of Na^+^, K^+^, and Ca^2+^ [16]. Ca^2+^, K^+^ and cyclic nucleotide channels are involved in early signaling during defense responses [17]. In rice, *OsCNGC14* and *OsCNGC16* promote tolerance to heat and chilling [18]. In Arabidopsis, compared with wild-type plants, mature A2 and A3 plants (*AtCNGC10* antisense lines) were shown to have altered K^+^ and Na^+^ concentrations in their shoots and were more sensitive to salt stress [19]. Moreover, compared with wild-type seedlings, *cngc3* mutant seedlings showed slightly enhanced growth in the presence of elevated NaCl or KCl concentrations [20]. Upon salinity, *AtCNGC19* and *AtCNGC20* were upregulated within hours; *AtCNGC19* and *AtCNGC20* aid plants by coping with toxic effects caused by salt stress [21]. These studies reveal that *CNGC* genes play important roles in plant biological processes and both provide an important direction and serve as a reference for further studies of this family of genes in response to abiotic stress.

In addition to being an essential oilseed crop species, cotton is one of the world’s most important fiber crop species [22, 23]. It is well known that the *CNGC* gene family exists in many species, but there is little research on this family in cotton. In our research, a total of 114 *CNGC* genes were identified from 4 cotton species (*Gossypium arboreum* (20)*, Gossypium raimondii* (20)*, Gossypium hirsutum* (38) and *Gossypium barbadense* (36)). To further study the potential role of cotton *CNGC* genes, we systematically analyzed the gene structure of, genetic relationships among and cis-acting elements in the promoters of 114 cotton *CNGC* genes. According to previous research on the role of *CNGC* genes in the response to abiotic stress (such as salt stress) in the model plant species Arabidopsis [19, 20], we speculate that cotton *CNGC* genes may play important roles in the response to salt stress and drought stress. Through a combination of transcriptome data and quantitative RT–PCR (qRT–PCR) results, the functions of the cotton *CNGC* genes were further analyzed. It was found that some *GhCNGC* genes were induced by salt and drought stress and responded to exogenous abscisic acid (ABA). In addition, virus-induced gene silencing (VIGS) experiments showed that the silencing of the *GhCNGC32* and *GhCNGC35* genes reduced the salt tolerance of cotton, and salt stress induced high expression of ABA-related genes. Taken together, these studies provide references for further studies on the regulatory mechanism of cotton *CNGC* genes with respect to salt stress tolerance.

## Results

### Genome-wide identification of *CNGC* genes in cotton species

After BLASTP alignment and confirmation of domain analysis via the CDD and SMART database, 169 putative *CNGC* genes were identified from the genomes of 7 species (Table S1). Of these genes, 114 *CNGCs* were found in *G. arboreum*, *G. raimondii*, *G. barbadense*, and *G. hirsutum* (20, 20, 36, and 38 genes respectively). The coding sequences of cotton *CNGCs* ranged from 1683 to 2313 bp, and consequently, the encoded protein sequences consisted of 560 to 770 amino acids, with an average of 716 amino acids (Table S2). In addition, the pI of CNGCs may play role in controlling the cationic channel. The pI of the CNGC proteins ranged from 6.53–9.62, of which 94.7% had a relatively high pI (> 8.8), and only 6 proteins had a low predicted pI value (between 6.5 and 7.52). The molecular weights among CNGC proteins ranged from 64.74–88.33 kDa. Subcellular localization showed that 109 of the 114 cotton CNGC proteins were located in the cell membrane.

### Phylogenetic analysis of the *CNGC* gene family

**Fig. 1:**
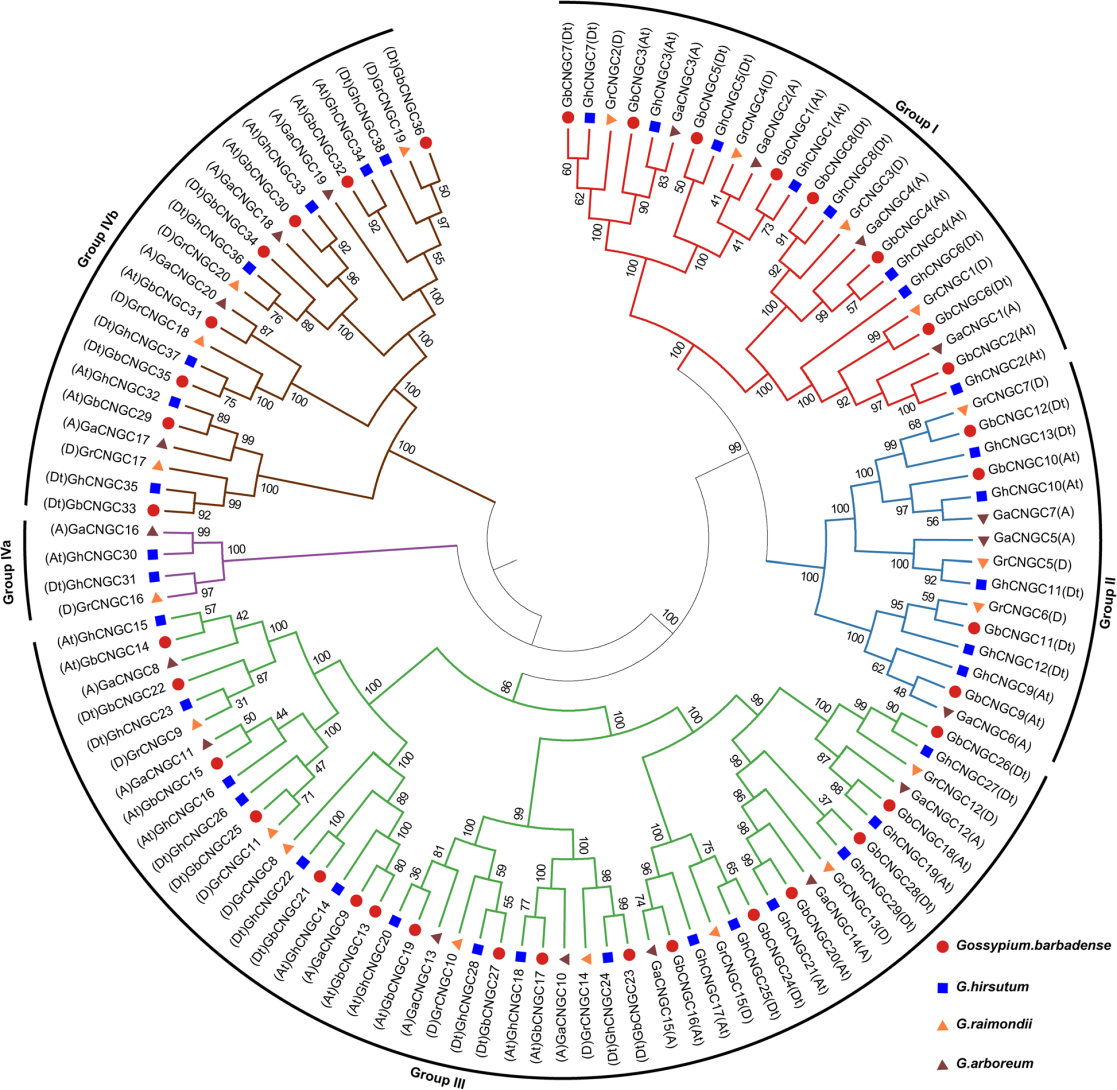
Phylogenetic relationships of *CNGC* genes derived from the genomes of two diploid (*G. arboreum* and *G. raimondii*) and two allotetraploid (*G. hirsutum* and *G. barbadense*) cotton species

To explore the phylogenetic relationship of *CNGC* genes among Gossypium species, the 114 cotton CNGC protein sequences and 55 other previously identified CNGC protein sequences from *A. thaliana*, *O. sativa*, and *P. trichocarpa* were used to construct a phylogenetic tree (Figure S1). The phylogenetic tree consisted of 169 CNGC proteins that were grouped into five subgroups: Group I, Group II, Group III, Group IVa, and Group IVb. To further analyze the phylogeny and selection pressure of *CNGC* genes in cotton species, the phylogenetic tree was reconstructed with only the 114 cotton CNGC sequences (Fig. 1). This cotton phylogenetic tree was also divided into 5 subfamilies, i.e., I, II, III, IVa, and IVb, that harbored 24, 15, 48, 4, and 23 genes, respectively.

### Gene structure and conserved motif analysis of cotton *CNGC* genes

**Fig. 2:**
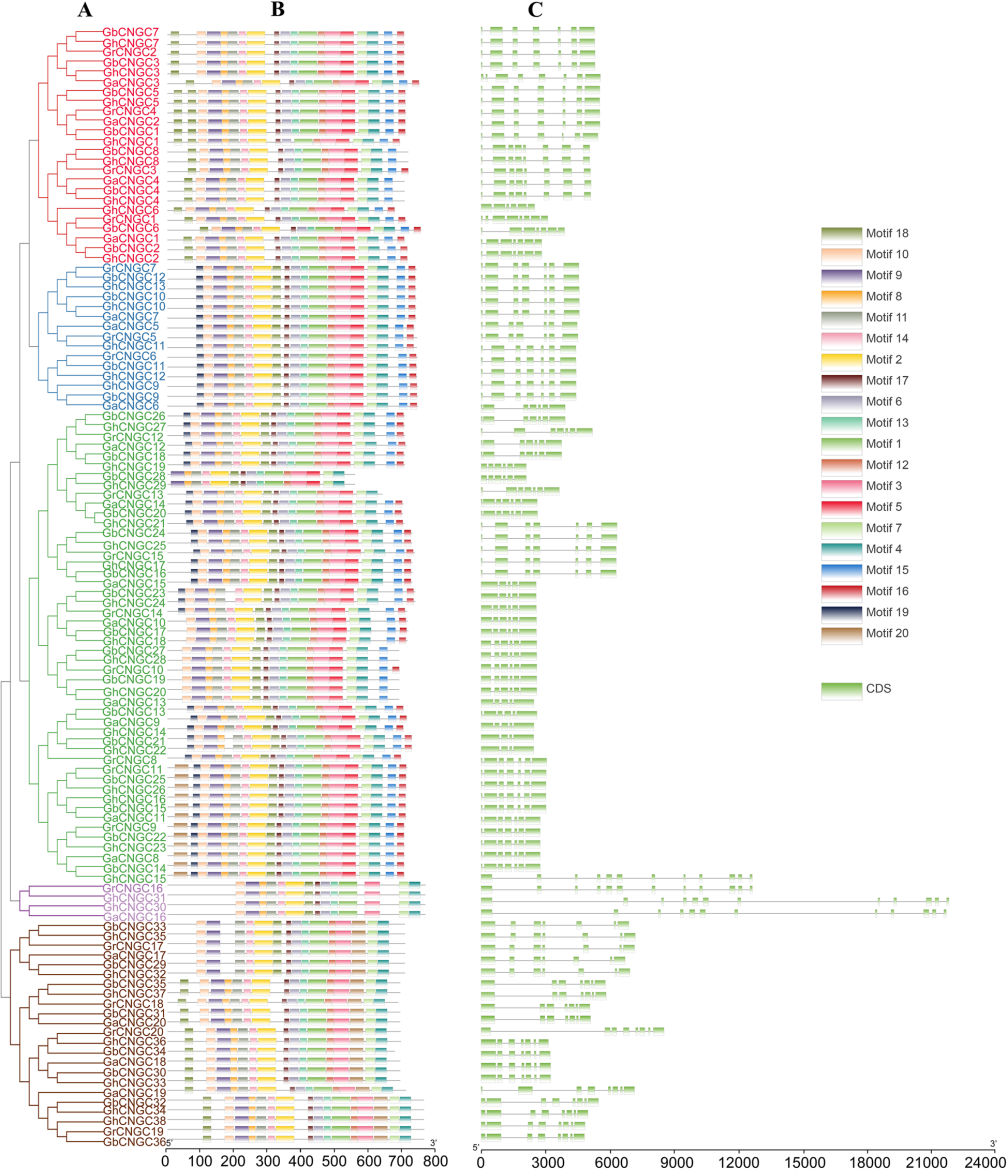
Phylogenetically aligned conserved motif and gene structure analysis of *CNGC* genes in *G. arboreum*, *G. raimondii*, *G. hirsutum*, and *G. barbadense*. **A** Phylogenetic tree of CNGC protein sequences; **B** Distribution of the predicted motifs in the CNGCs; **C** Exon–intron structure of *CNGC* genes in cotton

To investigate the diversity of gene structure among the 114 cotton *CNGCs*, the introns and exons of the cotton *CNGC* genes and the conserved motifs of the cotton CNGC proteins were analyzed (Fig. 2). Based on the clustering relationship, we observed that the members of the same clade displayed similar structures. The amino acid sequences of cotton CNGCs were analyzed by MEME software, and a total of 20 conserved motifs were found (Fig. 2B). Motif 9, motif 14, motif 2, motif 17, motif 6, motif 1, motif 3, motif 7, and motif 4 were distributed across all cotton CNGCs. Moreover, there were 3–11 introns within the cotton *CNGC* genes (Fig. 2C), which is similar to the numbers in Arabidopsis and rice, whose *CNGC* genes have 4–10 and 1–11 introns, respectively [24]. In the same subgroup, the number of exons and the type and spatial distribution of motifs in the *CNGCs* were highly similar, which indicates that *CNGC* genes in the same subfamily may have similar functions.

Multiple sequence alignment (MSA) showed that the cotton CNGC proteins had a unique motif, [LI]-X(2)-G-X-[FV]-X-G-[DE]-[DEH]-LL-X-W-X-L-X(10,22)-S-[TS]-X(5)-[ILV]-X(3)-EAF-X-L. Compared to the motif [LI]-X(2)-[GSE]-X-[VFIY]-XGX(0,1)-[DE]-LLXWX-[LQ]-X(10,20)-SX-[SAR]-X(7)-[VTI]-E-[AG]-FXL common in plants [25], the motif in cotton CNGCs contains a conserved (100%) glycine (G) in the PBC and a conserved (100%) aliphatic alanine (A) in the hinge region (Figure S2). Furthermore, the PBC and hinge regions within the CNBD of CNGC proteins exist only in plant CNGCs [5].

### Gene duplication and collinearity analysis of *CNGCs*

**Fig. 3:**
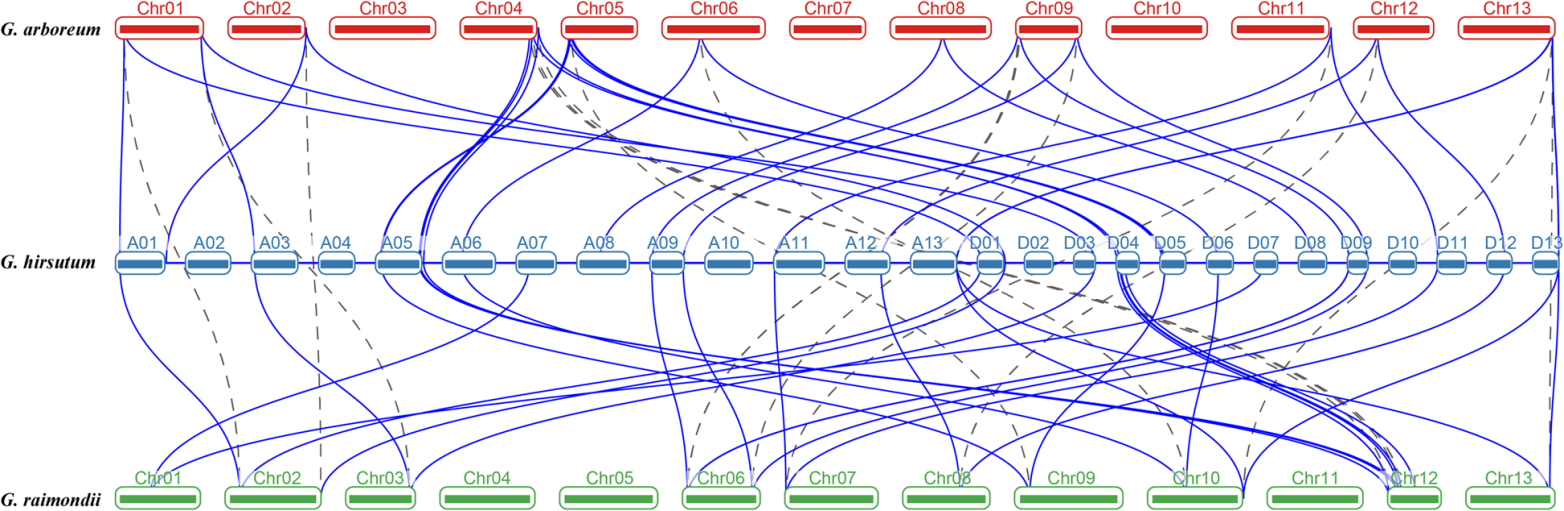
Collinearity analysis of *CNGC* genes between the tetraploid cotton species *G. hirsutum* and two diploid cotton species: *G. arboreum* and *G. raimondii*

To study the evolutionary relationship of *CNGC* genes in *G. hirsutum*, MCScanX software was used to analyze the collinearity of the At and Dt subgenomes of *G. hirsutum* and the corresponding ancestral A and D diploid genomes (Fig. 3). The results showed that the homologous gene pairs of *GhCNGC* genes were determined to be collinear between different genomes. Studies have revealed that whole-genome duplication (WGD) and tandem duplication have made important contributions to the evolution of gene families and the development of species diversity [26, 27]. Through collinearity analysis of homologous gene pairs of *G. hirsutum* and *G. barbadense*, 67 of the 74 genes were identified as resulting from WGD events (Table S3). No tandem repeats were detected, indicating that WGD is important drivers of *CNGC* gene family amplification. KaKs_Calculator2 was used to perform Ka/Ks analysis on the replicated gene pairs (Table S4). The Ka/Ks values of all the gene pairs were less than 1, indicating that the *CNGC* genes of cotton may have undergone purifying selection and were selected for in the process of evolution. It also can be seen that the Ks values of the A and D orthologs were approximately the same. The Ks values between A vs. At and D vs. Dt were very low (Figure S3).

### Analysis of cis-acting elements in *GhCNGCs*

Cis-acting elements usually play key roles in gene transcriptional regulation, such as responses to hormones and to abiotic stress [28]. Analysis of cis-acting elements in the *GhCNGC* genes showed that there were many types of abiotic stress response elements in the promoters, such as defense- and stress-, drought-, low-temperature- and salt stress-responsive cis-acting elements (Table S5). The numbers of cis-acting elements that respond to salt stress and drought stress were the highest—31 and 30, respectively. The response to abiotic stress is usually accompanied by hormone regulation [29, 30]. Analysis of the hormone-related cis-acting elements in *GhCNGCs* showed that the number of ABA-responsive cis-acting elements (60) was the highest (Table S5). ABA plays an important role in the regulatory response to salt and drought stress [30–32]. Based on the above results, we speculate that *GhCNGCs* may be closely related to the regulation of the response to abiotic stress (drought and salt stress).

### Analysis of expression patterns of *GhCNGC* genes

**Fig. 4:**
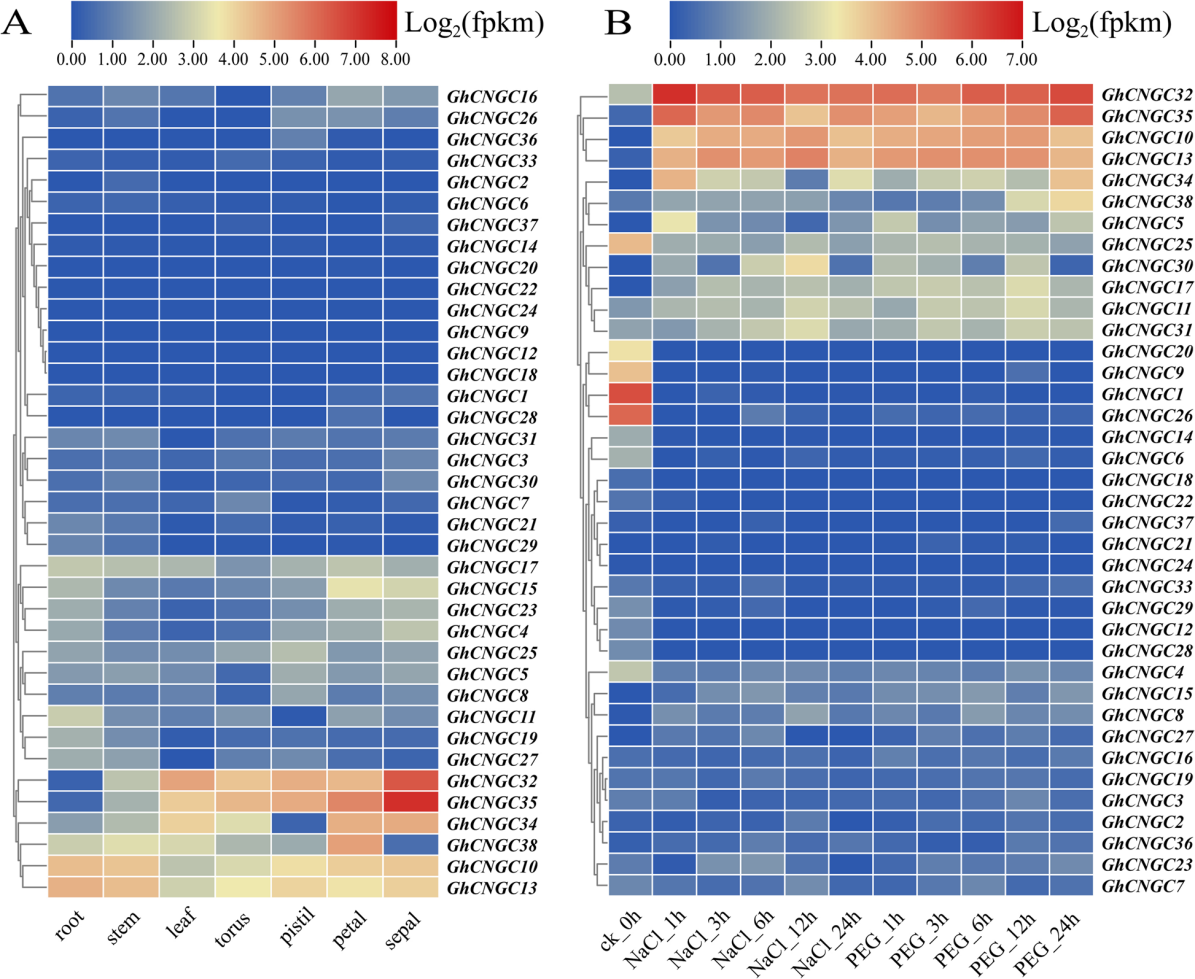
Expression profiles of *GhCNGCs* in different tissues (**A**) and in response to different stresses (**B**). The tissues or treatments are shown at the bottom, genes are shown on the right, and the phylogenetic relationships are shown on the left. The color scale in the upper part of the heatmap represents the FPKM values, which were standardized by the log_2_ method

To study the expression patterns of *GhCNGCs* in different tissues, the spatiotemporal expression patterns of these genes in the roots, stems, leaves, tori, pistils, petals and sepals within the transcriptome data were analyzed. Figure 4A shows that *GhCNGC10*, *GhCNGC13*, *GhCNGC32*, *GhCNGC34*, *GhCNGC35* and *GhCNGC38* are expressed in a variety of tissues, but the expression of other genes in these tissues was low. The expression levels of *GhCNGC32* and *GhCNGC35* were the highest in the sepals.

The results of the abiotic stress response element analysis showed that the *CNGC* family may be closely related to salt stress and drought stress responses. The transcriptome data concerning the response to salt and drought stress were used to analyze the expression patterns of *GhCNGC* genes (Fig. 4B). In response to salt and drought stress, *GhCNGC10*, *GhCNGC13*, *GhCNGC32* and *GhCNGC35* were significantly induced; *GhCNGC5*, *GhCNGC11*, *GhCNGC17*, *GhCNGC25*, *GhCNGC30*, *GhCNGC31*, *GhCNGC34* and *GhCNGC38* were also induced to varying degrees, but the degree of induction was relatively weak.

### Expression analysis of *GhCNGC* genes in response to ABA treatment

**Fig. 5:**
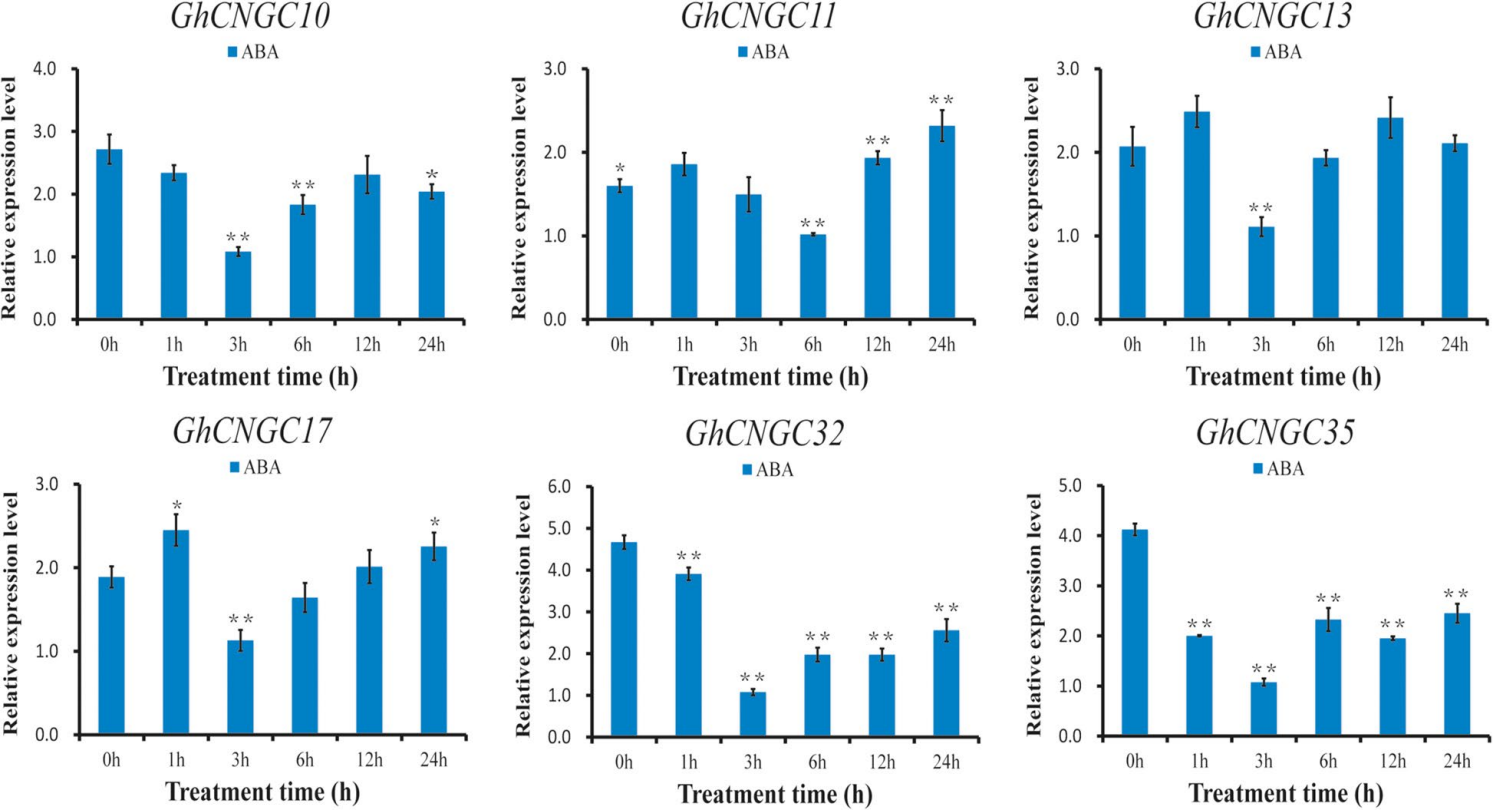
Relative expression levels of *GhCNGCs* in response to ABA treatment. The error bars show the standard deviations of three biological replicates

Based on the analysis of cis-acting elements and transcriptome data, *GhCNGCs* may be related to tolerance to salt and drought stress. ABA is an important hormone related to the regulation of salt and drought stress in plant [32], and the analysis of all *GhCNGC* promoters showed that the majority of cis-acting elements were related to the ABA response (Table S5). Six *GhCNGCs* were selected for stress treatment analysis in response to exogenous ABA (Fig. 5). The results showed that the six genes responded to treatment with ABA. Except for those of *GhCNGC11*, the relative expression levels of *GhCNGC10*, *GhCNGC13*, *GhCNGC17*, *GhCNGC32* and *GhCNGC35* were the lowest at 3 h after ABA treatment. According to the transcriptome analysis results of the expression patterns of genes in response to salt and drought stress, *GhCNGC32* and *GhCNGC35* were more responsive to stress than the other *GhCNGC* genes were (Fig. 5), and the expression levels of these two genes decreased significantly after ABA treatment.

### Silencing of *GhCNGC32* and *GhCNGC35* in cotton

**Fig. 6:**
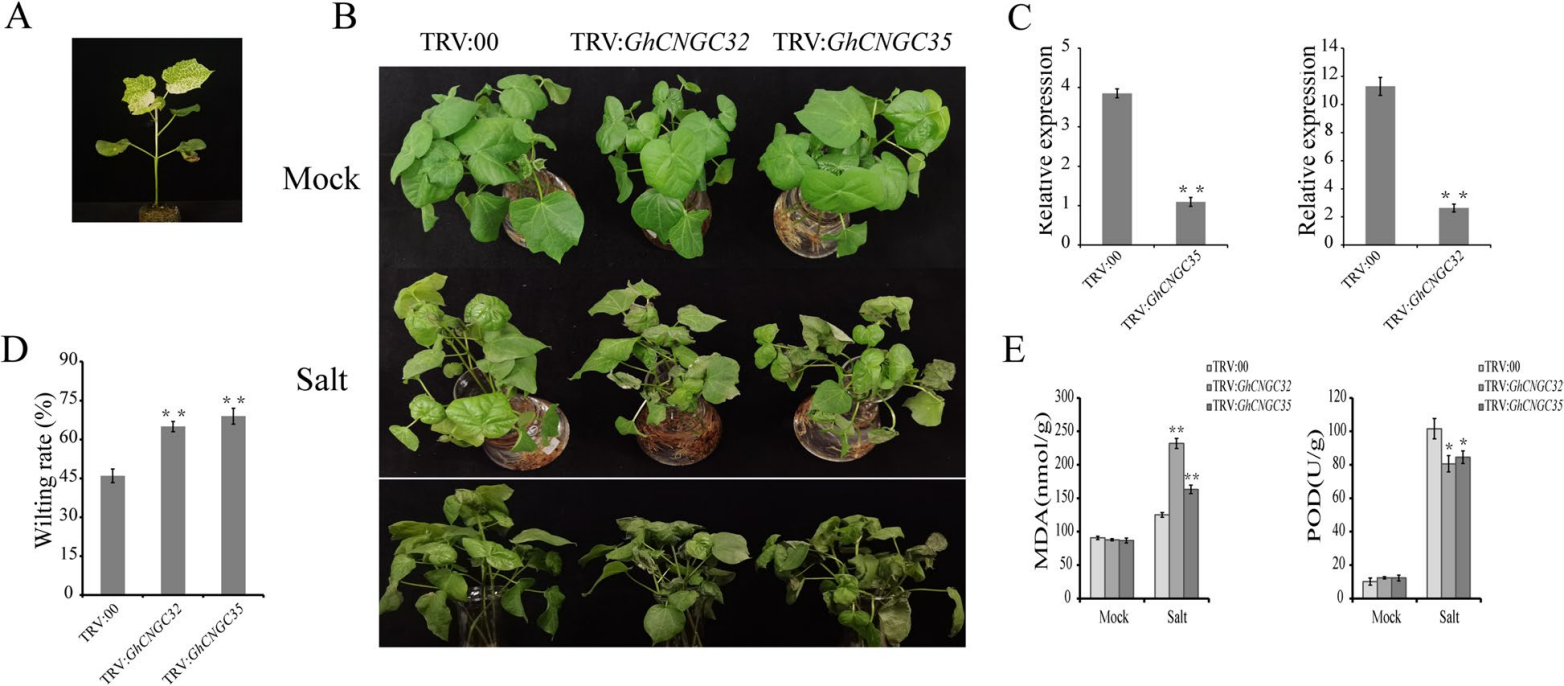
Silencing of *GhCNGC32* and *GhCNGC35* decreased resistance to salt stress in cotton. **A** Leaf whitening of TRV:*GhCLA1* (positive control); **B** Phenotypes of TRV:00, TRV:*GhCNGC32* and TRV:*GhCNGC35* under salt stress and normal conditions (Mock); **C** Relative expression levels of TRV:00, TRV:*GhCNGC32* and TRV:*GhCNGC35*; **D** Wilting rate; **E** MDA content and POD activity. The error bars indicate the standard deviations of the means of three independent experiments

Transcriptome data analysis showed that the homologous genes *GhCNGC32* and *GhCNGC35* were induced by salt stress (Fig. 4B). ABA is closely associated with the response to salt stress [31, 32]. The homologous genes *GhCNGC32* and *GhCNGC35* were substantially responsive to exogenous ABA, and the expression trend was similar in response to salt stress, with the expression level decreasing from 0–3 h; the lowest expression level occurred at 3 h but then increased (Fig. 5). To further verify the role of *GhCNGC32* and *GhCNGC35* in the response to salt stress, a VIGS experiment was performed. Approximately 15 days after the plants were infected, the control plants (TRV:*GhCLA1*) showed a significant albino phenotype (Fig. 6A), reflecting the validity of the VIGS experiment.

The expression levels of the *GhCNGC32* and *GhCNGC35* genes decreased significantly in the silenced plants, indicating that the genes were effectively silenced (Fig. 6C). After 3 days of high-concentration salt stress, all the plants showed a wilting phenotype, but the degree of wilting of the silenced plants was more severe than that of the control plants; moreover, the wilting rate of the silenced plants was significantly higher than that of the control plants (Fig. 6B and D). In addition, physiological indexes such as MDA content and POD activity under salt stress and normal conditions were measured (Fig. 6E). Under normal conditions, there was no significant difference in MDA content or POD activity between the control plants and silenced plants. Under salt stress, the MDA content of the silenced plants was significantly higher than that of the control plants, while POD activity decreased. These results suggest that *GhCNGC32* and *GhCNGC35* play roles in the regulation of salt tolerance in cotton and that silencing the *GhCNGC32* and *GhCNGC35* genes reduces cotton tolerance.

**Fig. 7:**
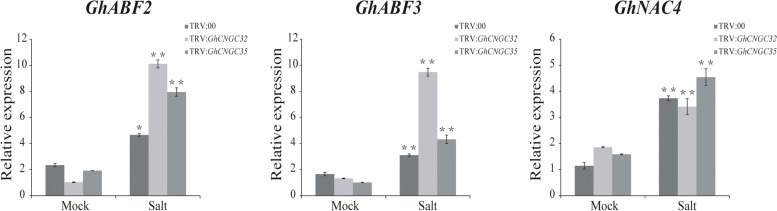
Relative expression of ABA-responsive genes (*GhABF2, GhABF3* and *GhNAC4*) in control plants and gene-silenced plants (TRV:*GhCNGC32* and TRV:*GhCNGC35*) under normal (mock) and salt stress conditions

As an important hormone closely related to salt stress, ABA plays a key role in salt stress regulation in plants [33, 34]. To further analyze the regulatory mechanism of salt tolerance in cotton, ABA-related genes (*GhABF2*, *GhABF3* and *GhNAC4*) were used targeted for VIGS [29, 35, 36]. Figure 7 shows that the expression levels of *GhABF2*, *GhABF3* and *GhNAC4* under salt stress were significantly higher than those under normal conditions. Under salt stress, the expression levels of *GhABF2* and *GhABF3* in the gene-silenced plants (TRV:*GhCNGC32* and TRV:*GhCNGC35*) were higher than those in the control plants (TRV:00).

## Discussion

*CNGC*s are cation channels with varying degrees of ion conduction selectivity. These channels allow monovalent and divalent cations to diffuse across membranes. Calmodulin (CaM) is considered to be a universal Ca^2+^ sensor in eukaryotes and can regulate the entry of *CNGC* cations [25]. Plant *CNGCs* have important functions in Ca^2+^ signal transduction, including pollen tube growth, heat sensitivity, and pathogen resistance [6, 37], and have been widely identified across the plant kingdom. *CNGC* gene families have been reported in many agriculturally important plant species [4, 38, 39]. However, the genome-wide identification and functional analysis of *CNGCs* in cotton have not yet been reported. In the past few years, the whole genomes of 4 cotton species have been completely sequences [40–44], and the resequencing of large cotton varieties has also been carried out, providing a basis for research on improving cotton functional genomics [23, 45–47].

### Phylogeny, gene structure, and expansion of *CNGC* genes in cotton

This research revealed 169 putative *CNGC* genes from the genomes of 7 species. Of these genes, 114 *CNGCs* were found in *G. arboreum*, *G. raimondii*, *G. barbadense*, and *G. hirsutum* (20, 20, 36, and 38 genes respectively). Allotetraploid cotton (*G. hirsutum*) is the result of the genomic hybridization and doubling of diploid A (*G. arboreum*) originating in Africa and diploid D (*G. raimondii*) originating in Mexico approximately 1–1.5 mya (Ks peaks at 0.005 and 0.008, respectively) [48], and gene loss is most likely an ongoing process in allotetraploid cotton [44]. This may result in the number of *CNGC* genes in allotetraploid cotton (*G. hirsutum* (38) and *G. barbadense* (36)) being less than the total number of *CNGC* genes in the two diploid cotton lines (*G. arboreum* (20) and *G. raimondii* (20)). Like in the cluster analysis results of Arabidopsis [21] and rice [24], the 114 cotton *CNGC* genes were divided into 5 groups (I, II, III, IVA, and IVB). Analysis of gene structure and protein sequences showed that *CNGC* gene members of each subfamily had similar gene structures, sequence lengths and motifs, which suggested that members of the *CNGC* family might exhibit relatively conserved functions during evolution. Most of the *CNGC* genes (94.7%) in cotton have a pI greater than 8.8. The pI and the charge of the protein are important for determining solubility, subcellular localization, and possible interactions. Moreover, there is a correlation between subcellular location and protein pI [49, 50]. According to reports, proteins that reside in cell membranes have more basic pIs (pI > 8.1) [51], and the regions where basic residues are located on both sides of the TM region play a role in the stability of proteins in the membrane [52]. Subcellular localization analysis showed that 109 out of 114 cotton *CNGC* genes were located on the cell membrane, further confirming that *CNGC* proteins are important ion channel proteins on the cell membrane [15, 16, 19]. The similarities and differences in the gene structure, domain and motifs of *GhCNGCs* may be related to the long evolutionary history of and gene duplication events in cotton [53]. Tandem duplication and WGD are the main driving forces of species evolution [54, 55]. The *CNGC* gene duplication types of *G. hirsutum* (38) and *G. barbadense* (36) showed that 67 of the 74 *CNGC* genes were amplified by WGD. Therefore, WGD was the main driving force in the process through which the *CNGC* gene family doubled from being diploid to allotetraploid in cotton.

### Functional analysis of the *GhCNGC32* and *GhCNGC35* genes involved in cotton salt tolerance

Studies have shown that *CNGCs* not only are involved in plant growth and development [20, 56] but also play an important role in the response to abiotic stress and signal transduction [4, 18, 19, 21, 39]. In *A. thaliana*, the *AtCNGC19* and *AtCNGC20* genes respond to salt stress and can help plants cope with toxic effects caused by salt stress [21]; mutant plants (*cngc3*) were more resistant to NaCl than wild-type plants were [20]. RNA sequencing (RNA-seq) data showed that *GhCNGC32* and *GhCNGC35* were significantly induced under salt and drought stress (Fig. 4B). ABA, a key hormone involved in abiotic stress regulation, plays an important role in plant regulation of the salt stress response. After exogenous ABA treatment, the homologous genes *GhCNGC32* and *GhCNGC35* were significantly induced, and on the whole, the expression tended to first decrease and then increase (Fig. 5).

VIGS (via the tobacco rattle virus (TRV) vector) has been widely used to study the function of genes related to the abiotic stress response in cotton. Cai et al. (2019) reported a detailed method involving the TRV-VIGS system for cotton gene function studies [57]. The homologous genes *GhCNGC32* and *GhCNGC35* were used for TRV-VIGS system studies. Compared with those in the control plants, the expression levels of the *GhCNGC32* and *GhCNGC35* genes in the transgenic plants were significantly decreased, and the TRV:*GhCLA1* plants (positive control) showed obvious albinism, indicating that gene expression was significantly inhibited and reflecting the validity of the VIGS experiment (Fig. 6A and C). After salt stress, the leaves of gene-silenced plants (TRV:*GhCNGC32* and TRV:*GhCNGC35*) were severely damaged, and the wilting degree was significantly higher than that of the control plants (TRV:00) (Fig. 6B and D). MDA content and POD activity, important indicators reflecting cell oxidative damage [58], were used to assess the physiology of the experimental plants. Abiotic stress, such as salt stress, usually disrupts normal cell homeostasis and leads to the accumulation of reactive oxygen species, which can be quenched by POD [59]. Under normal conditions, no significant differences in MDA content or POD activity were observed between the control plants (TRV:00) and gene-silenced plants (TRV:*GhCNGC32* and TRV:*GhCNGC35*). After salt stress, the MDA content and POD activity of the gene-silenced plants significantly increased. The MDA content accumulated more in the gene-silenced plants (TRV:*GhCNGC32* and TRV:*GhCNGC35*) than in the control plants (TRV:00), while the POD activity significantly decreased in the gene-silenced plants (TRV:*GhCNGC32* and TRV:*GhCNGC35*) (Fig. 6E). Taken together, these results indicate that *GhCNGC32* and *GhCNGC35* play a positive regulatory role in the response to salt stress in cotton.

Studies have reported that ABA plays a crucial role in the response to abiotic stress and is a central regulator of the abiotic stress response [60, 61], leading to significant changes in gene expression and adaptive physiological responses [34]. Salt stress can induce the ABA signaling regulatory mechanism, which plays an important role in the regulation of plant salt tolerance [62, 63]. The qRT–PCR results showed that the expression levels of ABA-related genes (*GhABF2*, *GhABF3* and *GhNAC4*) in both the control (TRV:00) and gene-silenced plants (TRV:*GhCNGC32* and TRV:*GhCNGC35*) were significantly higher than those under normal conditions after salt stress, and the expression levels of *GhABF2* and *GhABF3* in the gene-silenced plants (TRV:*GhCNGC32* and TRV:*GhCNGC35*) were higher than those in the control plants (TRV:00) (Fig. 7). The upregulated expression of *GhABF2*, *GhABF3* and *GhNAC4* may play a certain role in the regulation of salt tolerance in cotton.

## Conclusions

In this study, 114 cotton *CNGC* genes were identified in *G. arboreum*, *G. raimondii*, *G. hirsutum*, and *G. barbadense* (20, 20, 38, and 36, respectively). The gene structure, gene duplication type and collinear relationship between tetraploid species and their diploid ancestors were systematically studied. RNA-seq data showed that *GhCNGC* genes responded to salt and drought stress, and VIGS experiments confirmed that the silencing of *GhCNGC32* and *GhCNGC35* decreased the salt tolerance of cotton. The upregulation of ABA-related gene expression under salt stress indicates that salt stress may trigger the regulatory mechanism of ABA signaling, regulating cotton salt tolerance. Further study of the steady-state regulation of ABA under salt stress will help to clarify the comprehensive effects of ABA on the regulation of salt tolerance in cotton.

## Methods

### Identification and alignment of ***CNGC*** family genes

Published genomes and protein sequences from seven species were downloaded and used as database sources for identifying *CNGC* genes: *Arabidopsis thaliana* from The Arabidopsis Information Resource 10 (TAIR10; http://www.arabidopsis.org/); *Oryza sativa* from IRGSP-1.0 (https://rapdb.dna.affrc.go.jp/); *Populus trichocarpa* from Phytozome (https://phytozome.jgi.doe.gov/pz/portal.html#!info?alias=Org_Ptrichocarpa); and *G. arboreum*, *G. raimondii*, *G. hirsutum*, and *G. barbadense* from CottonGen (https://www.cottongen.org/data/download/). Initially, the 20 *CNGCs* that were identified in *A. thaliana* were obtained from TAIR [64] and used to query the protein database by BLASTP, with the e-value of 1e-10 used as a cutoff value. After removing redundant sequences, the protein sequences were submitted to the Conserved Domains Database (CDD) [65], SMART database (http://smart.embl-heidelberg.de/), and Pfam database (https://pfam.xfam.org/) were used for domain analysis. These sequences contained a cyclic nucleotide-monophosphate binding domain (cNMP), a TM, an ion transport protein (ITP) domain, or an Ion_trans family (PF00520) domain. Finally, these genes were aligned against 20 *AtCNGC* and 16 *OsCNGC* sequences that were identified by Xu [66] via the ClustalW program [67], with the default parameters. A neighbor-joining tree was constructed with the MSA file using MEGA 6.06 [68] with the p-distance model and 1000 bootstrap replicates. Proteins that contained a PBC and hinge region with the CNBD motif for plant *CNGCs* were recognized as *CNGC* members [13].

### Multiple sequence alignment (MSA) and phylogenetic tree construction

After obtaining the 169 identified *CNGC* gene sequences, the ClustalW program [67] was used for MSA, which was then used for phylogenetic analysis. A neighbor-joining tree was constructed with the MSA file using MEGA 6.06 [68] with the p-distance model and 1000 bootstrap replicates. The phylogenetic tree of the *CNGC* family was ultimately divided into five groups on the basis of the *AtCNGC* gene family [21].

### Gene structure, protein sequence and conserved domain analysis

Structural information of the cotton *CNGC* genes was retrieved from the GFF3 file containing genome information, and the exon/intron structures were generated with Structure Display Server (GSDS) 2.0 (http://gsds.cbi.pku.edu.cn/). The motifs of the CNGC proteins were predicted by the Multiple Expectation Maximization for Motif Elicitation (MEME) [69] website; the default parameters were used, with the exception that the number of motifs was set to 10. The CNGC protein isoelectric points (PIs) and chemical properties were analyzed by ExPASy (https://www.expasy.org). Afterward, using ClustalX 2.1 (http://www.clustal.org/clustal2) software, we performed a multiple alignment of protein sequences and used GeneDoc 2.7 (https://github.com/karlnicholas/GeneDoc) software to extract conserved domain sequences. Finally, we used WebLogo online software [70] to show the conservation of the amino acid sequence of the consensus CNGC conserved region in *G. hirsutum*.

### Retrieval and analysis of *GhCNGC* promoter sequences

DNA sequences comprising 2000 bp upstream of the start codon (ATG) of the genes *CNGC* were determined and queried within the PlantCARE online database to identify cis-acting elements in the promoter regions [71]. TBtools was subsequently used to visualize the distribution of cotton *CNGC* cis-acting elements [72].

### Collinearity and selection pressure analysis

An all-versus-all BLASTP search (e-value < 1e-5) was performed using the protein sequences from the four aforementioned Gossypium species. Then, the MCScanX program [73] was used to detect orthologous and paralogous gene pairs and explore the duplication types of *CNGC* genes. The collinearity of *CNGCs* between two diploid species, *G. hirsutum* and *G. barbadense*, was visualized using TBtools [72], and the collinearity between two tetraploid species, *G. hirsutum* and *G. barbadense*, was represented by a circular map generated by the Circos program [74]. ParaAT and KaKs_Calculator 2.0 [75] software were used to calculate the nonsynonymous mutation rate (Ka) and the synonymous mutation rate (Ks) of the replication gene pairs in *G. arboreum*, *G. raimondii*, *G. hirsutum*, and *G. barbadense*.

### Transcriptome data analysis and qRT–PCR experiments

To understand the expression of *CNGC* genes in *G. hirsutum*, transcriptome datasets were obtained from the Sequence Read Archive (SRA) dataset of the NCBI database via accession number PRJNA490626 [27]. HISAT2 [76] was used for alignment to the reference genome, and Cufflinks [77] was used to calculate expression levels (fragments per kilobase of transcript per million mapped reads (FPKM)). HemI 1.0.3.7 software was used for subsequent visualization of the results [78]. An ABI 7500 Real-time PCR system (Applied Biosystems) was used to carry out qRT–PCR experiments. For each sample, three biological replications were included to obtain reliable results, and the data were calculated according to the 2^−ΔΔCt^ formula [79]. Gene-specific primers for qRT–PCR were designed by Oligo 7 [80] (Table S1).

### Plant materials and treatments

The upland cotton cultivar “Texas Marker 1” (TM-1) was planted in a climate-controlled chamber with a light/dark cycle of 16 h at 28 °C/8 h at 22 °C. When the third true leaf had unfolded (at approximately four weeks after planting), the leaves were sprayed with 200 mM ABA. The leaves of three seedlings were collected at 0 h, 1 h, 3 h, 6 h, 12 h and 24 h after treatment. All the samples were immediately frozen in liquid nitrogen and stored at -80 °C.

### Silencing of *GhCNGC32* and *GhCNGC35* genes in cotton

The web-based SGN VIGS tool was used to design silenced fragments of *GhCNGC32* and *GhCNGC35* [81]. These fragments were then ligated into a pTRV2 (pYL156) vector. The primers used for vector construction are listed in Table S1. The recombinant vector was transformed into *Agrobacterium tumefaciens* LBA4404. LBA4404 bacterial solutions carrying pTRV2 (empty vector), pTRV2-*GhCNGC32*, pTRV2-*GhCNGC35*, pTRV2-CLA1 (Cloroplastos alterados 1, positive control) and pTRV1 (pYL192) were injected into the cotyledons of TM-1. After 24 h of dark treatment, the cotton seedlings were moved to a greenhouse with 16 h of light/8 h of darkness, allowed to grow unabated for approximately 15 days and then treated with 300 mM NaCl. The wilting rate of cotton was calculated as the percentage of wilted plants among the total number of stressed plants. Sample (leaf) powder comprising a mixture of at least 15 cotton plants was taken to determine the malondialdehyde (MDA) and peroxidase (POD) activity (Solarbio, Beijing, China). The specific steps were performed according to the manufacturers’ instructions. Three biological replications were included.

## Supplementary Information

**Additional file 1: Figure S1.** Phylogenetic tree showing the 169 *CNGC* genes from 7 species: *A. thaliana*, *O. sativa*, *P. trichocarpa*, *G. arboreum*, *G. hirsutum*, *G. raimondii*, and *G. barbadense*.

**Additional file 2: Figure S2.** The multiple sequence alignment of the *CNGC* gene family.

**Additional file 3: Figure S3.** Ks distribute of *CNGC* gene pairs of four cotton species.

**Additional file 4: Table S1.** Numbers of *CNGC* genes in each group of seven plant species.

**Additional file 5: Table S2.** Detailed physicochemical characteristics of CNGC proteins of *G. hirsutum*, *G. barbadense*, *G. arboreum* and *G. raimondii*.

**Additional file 6: Table S3.** Duplicate type of *CNGCs* in *G. hirsutum* and *G. barbadense*.

**Additional file 7: Table S4.** Ka/Ks ratios of *CNGC* gene pairs of four cotton species.

**Additional file 8: Table S5.** Statistical results of cis-acting response elements in the promoter segments of *GhCNGCs*.

**Additional file 9: Table S6.** List of the primers used in this study.

## Data Availability

The gene sequences of *Arabidopsis thaliana* in this study are available in TAIR (http://www.arabidopsis.org/); The gene sequences of *Oryza sativa* are available in IRGSP-1.0 (https://rapdb.dna.affrc.go.jp/); The gene sequences of *Populus trichocarpa* are available in Phytozome (https://phytozome.jgi.doe.gov/pz/portal.html#!info?alias=Org_Ptrichocarpa); The gene sequences of *G. arboreum*, *G. raimondii*, *G. hirsutum*, and *G. barbadense* can be downloaded from CottonGen (https://www.cottongen.org/data/download/). The transcriptome datasets of cotton can be downloaded from the NCBI website under the BioProject PRJNA490626 (https://www.ncbi.nlm.nih.gov/bioproject/PRJNA490626). All data supporting the conclusions of this article are included in the article and its additional files.

## Abbreviations

CNGC: Cyclic nucleotide-gated ion channels
VIGS: Virus-induced gene silencing
WGD: Whole-genome duplication
ABA: Abscisic acid
