# Supplementary figures and images for: Systematic analysis of CNGCs in cotton and the positive role of GhCNGC32 and GhCNGC35 in salt tolerance
Source: BMC Genomics. 2022 Aug 5;23:560. doi: 10.1186/s12864-022-08800-5 (PMC9356423; doi:10.1186/s12864-022-08800-5)

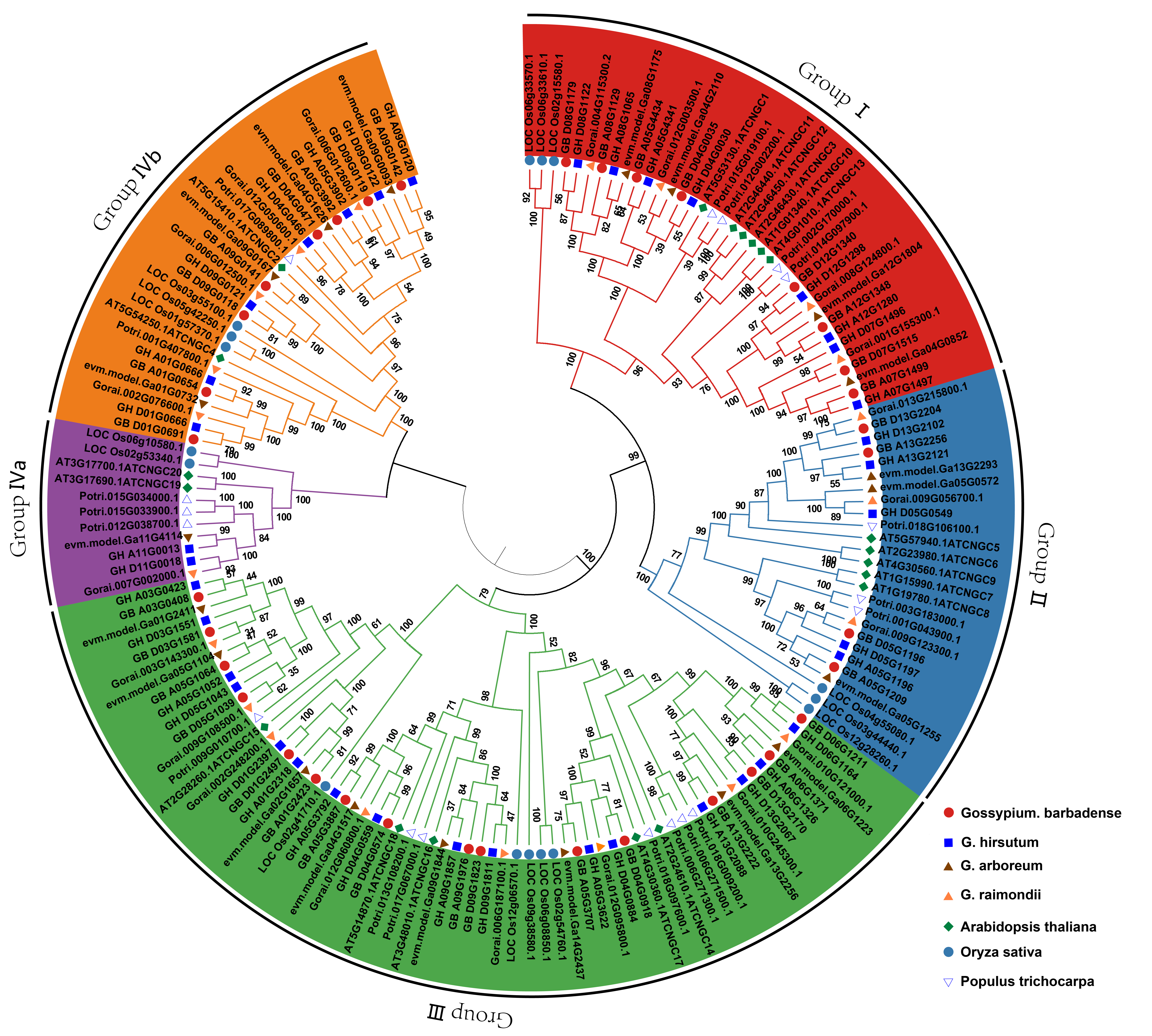

Supplement: Supplementary file 1 — Additional file 1: Figure S1. Phylogenetic tree showing the 169 CNGC genes from 7 species: A. thaliana, O. sativa, P. trichocarpa, G. arboreum, G. hirsutum, G. raimondii, and G. barbadense. [file 12864_2022_8800_MOESM1_ESM.tif]

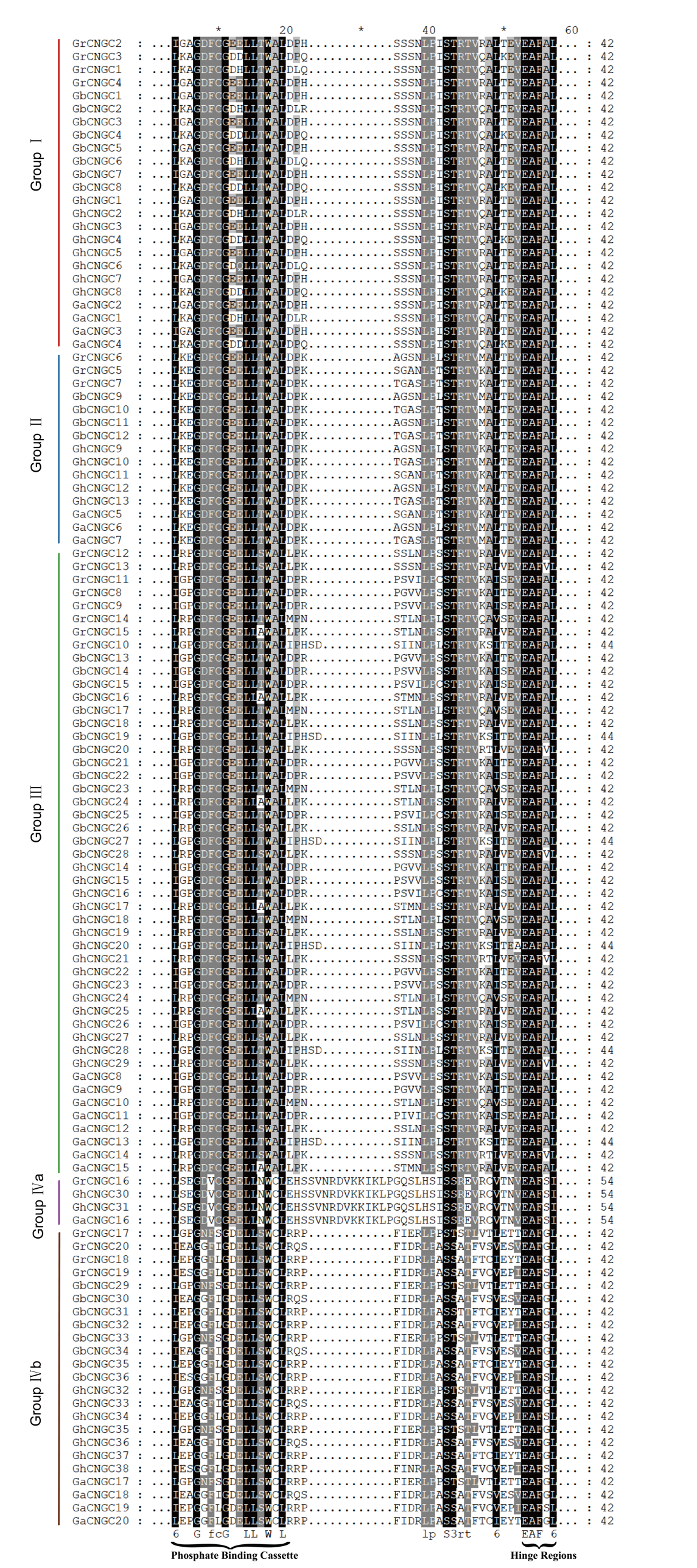

Supplement: Supplementary file 2 — Additional file 2: Figure S2. The multiple sequence alignment of the CNGC gene family. [file 12864_2022_8800_MOESM2_ESM.tif]

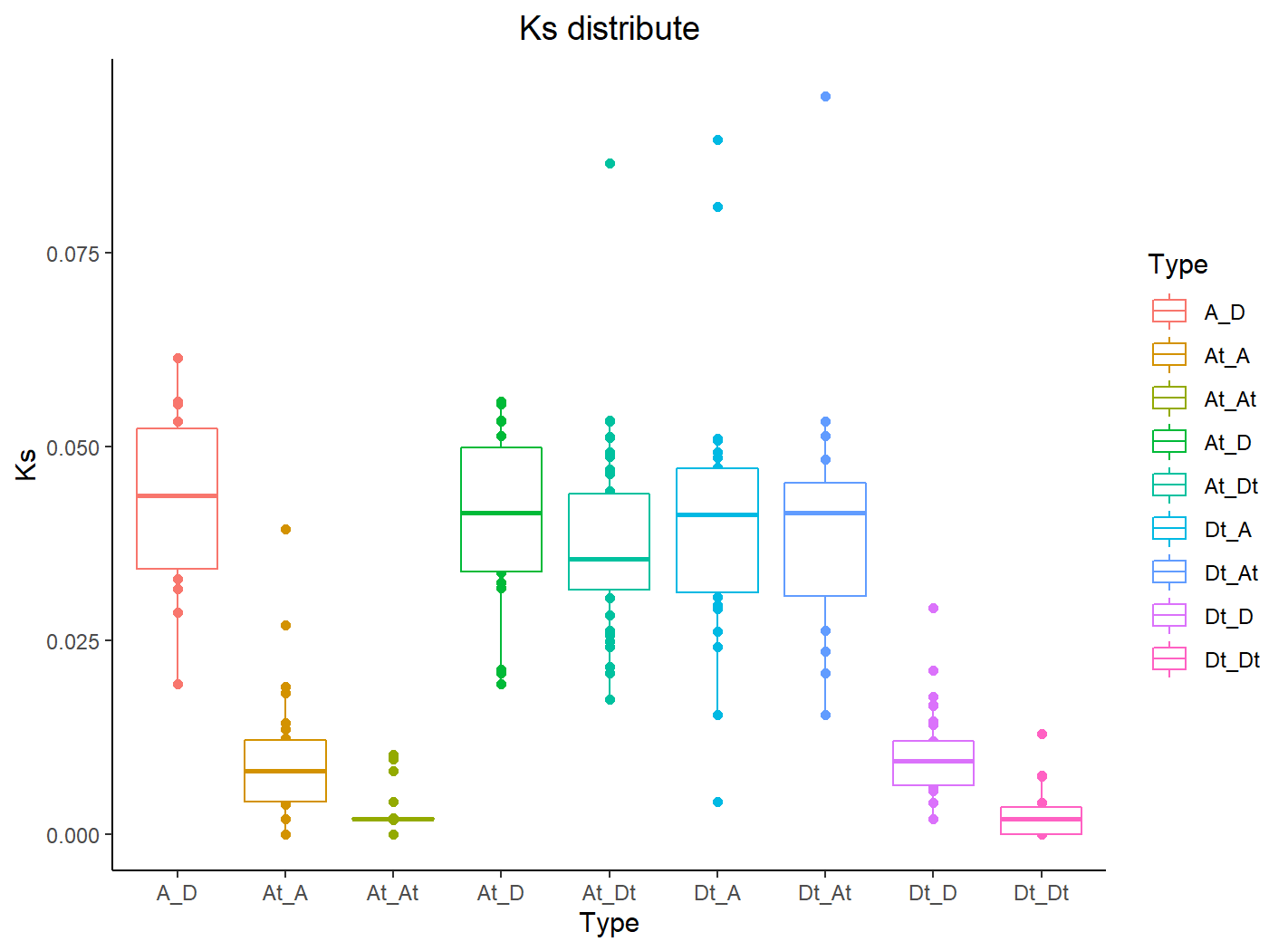

Supplement: Supplementary file 3 — Additional file 3: Figure S3. Ks distribute of CNGC gene pairs of four cotton species. [file 12864_2022_8800_MOESM3_ESM.tif]
